# MSX2 suppression through inhibition of TGFβ signaling enhances hematopoietic differentiation of human embryonic stem cells

**DOI:** 10.1186/s13287-020-01653-3

**Published:** 2020-04-05

**Authors:** Hongtao Wang, Mengge Wang, Yu Wang, Yuqi Wen, Xiaoyuan Chen, Dan Wu, Pei Su, Wen Zhou, Lihong Shi, Jiaxi Zhou

**Affiliations:** 1 State Key Laboratory of Experimental Hematology, National Clinical Research Center for Blood Diseases, Institute of Hematology & Blood Diseases Hospital, Chinese Academy of Medical Sciences & Peking Union Medical College, Tianjin, 300020 China; 2 Center for Stem Cell Medicine, Chinese Academy of Medical Sciences & Department of Stem Cells and Regenerative Medicine, Peking Union Medical College, Tianjin, 300020 China; 3 Department of Hematology, Xiangya Hospital, Central South University, Changsha, Hunan China; 4 Key Laboratory of Carcinogenesis and Cancer Invasion, Ministry of Education; Key Laboratory of Carcinogenesis, National Health and Family Planning Commission; Cancer Research Institute, School of Basic Medical Science, Central South University, Changsha, Hunan China

**Keywords:** MSX2, TGF-β signaling, Endothelial to hematopoietic transition, Human embryonic stem cells, Hematopoietic differentiation

## Abstract

**Background:**

Strategies of generating functional blood cells from human pluripotent stem cells (hPSCs) remain largely unsuccessful due to the lack of a comprehensive understanding of hematopoietic development. Endothelial-to-hematopoietic transition (EHT) serves as the pivotal mechanism for the onset of hematopoiesis and is negatively regulated by TGF-β signaling. However, little is known about the underlying details of TGF-β signaling during EHT.

**Methods:**

In this study, by applying genome-wide gene profiling, we identified muscle segment homeobox2 (MSX2) as a potential mediator of TGF-β signaling during EHT. We generated MSX2-deleted human embryonic stem cell (hESC) lines using the CRISPR/Cas9 technology and induced them to undergo hematopoietic differentiation. The role of MSX2 in hematopoiesis and functional regulation of TGFβ signaling in EHT was studied.

**Results:**

We identified MSX2 as a novel regulator of human hematopoiesis. MSX2 deletion promotes the production of hematopoietic cells from hESCs. Functional and bioinformatics studies further demonstrated that MSX2 deletion augments hematopoietic differentiation of hESCs by facilitating EHT. Mechanistically, MSX2 acts as a downstream target of TGFβ signaling to mediate its function during EHT.

**Conclusions:**

Our results not only improve the understanding of EHT, but may also provide novel insight into the efficient production of functional blood cells from hPSCs for regenerative medicine.

## Background

Human embryonic stem cells (hESCs), generated from the inner cell mass of human embryos, have the capacity of self-renewal and multi-lineage differentiation, thus offering an invaluable tool for dissecting early human hematopoietic development and the ex vivo production of hematopoietic stem cells (HSCs) and functional blood cells for therapies of various hematologic disorders [1–3]. However, it remains a great challenge to generate HSCs with robust multi-lineage engraftment potential and infusion dosage levels of functional blood cells from hESCs, mainly because of the lack of understanding of the underlying mechanisms controlling hematopoietic development [4].

hESC hematopoietic differentiation goes through three main stages, including mesoderm induction, the emergence of hemogenic endothelium progenitors (HEPs), and generation of hematopoietic progenitor cells (HPCs), which largely mimic embryonic hematopoietic development in vivo [5]. HPCs emerge from HEPs through the process of endothelial-to-hematopoietic transition (EHT), which serves as a vital mechanism for the initiation of hematopoiesis and is tightly controlled by several signaling pathways [6, 7]. Retinoic acid (RA) signaling reportedly enhances hESC hematopoietic differentiation by facilitating EHT [8]. Recent studies also revealed the critical role of HOXA family members in mediating the function of RA signaling in hematopoietic differentiation [8, 9]. In addition, a number of studies have demonstrated that the NOTCH signaling pathway promotes EHT by activating HES1 both in vitro and in vivo [10–14]. In contrast, TGFβ signaling exerts a negative effect during EHT [15–18]. Elevated expression of TGFβ signaling components has been identified in endothelial cells and decreased during EHT in mouse embryos [15]. Activation of TGFβ signaling completely abolishes the generation of HPCs from HEPs, while TGFβ inhibition promotes the transition—a response conserved in both mouse and human embryonic stem cells [15–18]. Despite its well documented vital role in EHT, little is known about *how* TGFβ signaling exerts its function during this process.

Aside from the cytoplasmic signaling pathways, transcription factors also play a key role in the regulation of EHT [6, 7]. RUNX1 and GATA2 are indispensable for EHT both in vitro and in vivo [19, 20]. Overexpression of SCL/TAL1 severely promotes the emergence of blood cells from endothelial cells during hESC hematopoietic differentiation [21], while knockout of MEIS2 severely suppresses hESC hematopoietic differentiation by impairing EHT [22]. Meanwhile, there are also transcription factors that play negative roles in EHT. For example, HOXA3 activates the endothelial program by repressing the expression of RUNX1, leading to the impairment of EHT [23]. Additionally, SOX17 prevents EHT by maintaining the endothelial identity of cells, and SOX17 downregulation is required for the emergence of blood cells [24]. In addition, suppression of ID1 or ID3 augments the generation of hematopoietic cells from HEPs during hematopoietic differentiation of hESCs [25]. A combined manipulation of the vital transcription factors is effective in directing the induction of hematopoietic cells from somatic cells and human pluripotent stem cells (hPSCs). For instance, fibroblasts can be reprogrammed into hematopoietic stem and progenitor cells (HSPCs) with short-term engraftment by the use of a series of EHT-regulating factors including Runx1c, Gata2, and Scl [26]. A separate study showed that 7 transcription factors including RUNX1 are sufficient to convert hPSC-derived HEs into transplantable HSPCs [27]. Therefore, identification of novel transcription factors controlling EHT not only should advance our understanding of hematopoietic development but also will help to establish potential new strategies of HSC generation from hPSCs.

Muscle segment homeobox2 (MSX2), a homeobox-containing transcription factor, is implicated in organogenesis and the development processes of several types of tissues, including craniofacial tissues, limb, heart, and neural crest derivates [28–30]. However, whether MSX2 plays a role in hematopoietic development is still unclear. In the current study, by using genome-wide transcriptomic analysis and the CRISPR-Cas9 technology, we identified MSX2 as a novel regulator of EHT that mediates the function of TGFβ signaling. Our results unveiled the key role of MSX2 in hematopoietic differentiation and established a novel signaling link between key cytoplasmic signaling pathways and transcription factors during hematopoietic differentiation of hESCs. Therefore, our findings may facilitate the development of new efficient strategies to produce large-scale functional blood cells from hPSCs.

## Materials and methods

### hESC cultivation and hematopoietic differentiation

**Fig. 1:**
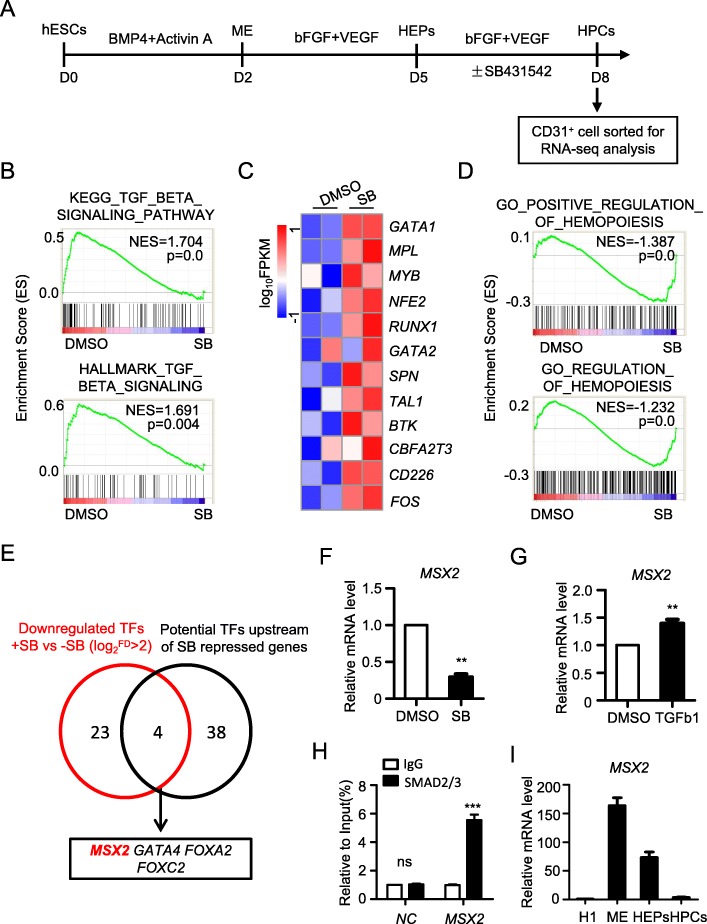
MSX2 is suppressed upon inhibition of TGFβ signaling during hematopoietic differentiation of hESCs. **a** Schematic overview of hESC hematopoietic differentiation using a chemically defined system. SB431542 (SB) was added during days 5–8, and RNA-seq was performed on CD31^+^ cells at day 8. **b** GSEA of TGFβ signaling-associated gene sets with or without SB treatment. **c** Heatmap of hematopoiesis-related signature genes with or without SB addition. **d** GSEA of hematopoiesis-associated gene sets with or without SB treatment. **e** Schematic diagram showing the strategy of screening potential mediators of TGFβ signaling in hematopoietic differentiation. The red circle represents downregulated TFs upon SB treatment (also see Fig. S1A). The black circle represents potential TFs upstream of SB-repressed genes analyzed by using Enrichr (also see Fig. S1B). **f** The real-time PCR analysis of *MSX2* expression in cells at day 8 of hematopoietic differentiation with or without SB treatment. **g** The real-time PCR analysis of *MSX2* expression in cells at day 8 of hematopoietic differentiation with or without TGFβ1 treatment. Relative expression is normalized to the level (= 1) of Actin. Results are shown as means ± SD (*n* = 3). **h** ChIP-qPCR analysis of SMAD2/3-responsive elements on promoters of MSX2 in H1-derived cells. Non-specific IgG was used as isotype control. All values are normalized to that of their corresponding input samples. Results are shown as means ± SD (*n* = 3). **i** Real-time PCR analysis of *MSX2* expression in undifferentiated hESCs, MEs (APLNR^+^), HEPs (CD31^+^CD34^+^), and HPCs (CD43^+^) generated from hematopoietic differentiation of hESCs. Relative expression is normalized to the level (= 1) of undifferentiated hESCs. Results are shown as means ± SD (*n* = 3). **P < 0.01 and ***P < 0.001

To maintain the pluripotent state, H1 hESCs (WiCell Research Institute, Madison, WI) were cultivated in mTeSR1 (Stem cell Technology) on plates coated with Matrigel (Corning) as described previously [31]. Hematopoietic differentiation from hESCs in a chemically defined system was performed as described before [22]. With Accutase dissociation, hPSCs were seeded as single cells onto growth factor-reduced (GFR) Matrigel (Corning)-coated plates at the density of 3.5 × 10^4^ cells/mL. Hematopoietic differentiation was induced 24 h later by medium changing with Custom mTeSR1 (Stem cell Technology) supplemented with a combination of different cytokines as shown in Fig. 1a. SB431542 (20 μM) (STEMGENT) were treated during days 5–7. For the further generation of CD45^+^ hematopoietic cells, differentiated cells at day 8 were dissociated and seeded into a suspension culture for another 6 days in low-attachment plates as described previously [22]. All cytokines used were from Peprotech.

### Establishment of MSX2 knockout hESC lines with CRISPR/Cas9

Single guide RNAs (sgRNAs) targeting the second exon of MSX2 were designed utilizing the CRISPR Design Tool (http://tools.genome-engineering.org) [32]. The oligonucleotides were annealed and cloned into the CRISPR-Cas9-Lenti-V2 vector as described before [31]. Lentivirus carrying the constructed vector was packaged and delivered into H1 hESCs. After puromycin selection, the infected cells were then dissociated into single cells, and small colonies were selected. The deletion of the MSX2 gene was verified by using sequencing. The sgRNA sequences and primers for MSX2 genotyping are listed in Supplemental Table S1.

### Establishment of MSX2 inducible knockdown hESC lines

We constructed MSX2 shRNAs or a scramble shRNA (shScramble) into a doxycycline (DOX)-inducible pLKO-Tet-On vector and established the inducible knockdown H1 hESC lines. Briefly, shRNA-containing lentivirus was packaged from HEK293T cells and then infected H1 cells which were seeded as single cells onto Matrigel-coated plates at the density of 1 × 10^5^ cells/mL 24 h before infection. After puromycin selection, the infected cells were dissociated into single cells and small colonies were picked for selections. Those sequences for MSX2 shRNAs and shScramble are listed in Supplemental Table S1.

### Flow cytometry and cell sorting

The differentiated cells were disassociated with Accutase and stained with fluorescein-conjugated antibodies after resuspension with 0.1% BSA. After 30 min of incubation, cells were washed and filtered through a 70-μm cell strainer to acquire single-cell suspension. Before analysis, DAPI was added to detect and exclude dead cells. Detailed information for the antibodies is listed in Supplemental Table S2. Flow cytometry analysis was performed using a FACS CantoII flow cytometer (BD Biosciences), and cell sorting was performed using FACS Aria III sorter (BD Biosciences).

### Colony-forming unit assay

5 × 10^4^ hematopoietic cells derived from WT and MSX2^−/−^ H1 cells were replated into methylcellulose H4435 (Stem Cell Technologies). After 2 weeks differentiating at 37 °C in 5% CO_2_ condition, distinct hematopoietic lineage colonies were counted and scored based on the standard morphological criteria.

### Immunofluorescence

Differentiated cells were fixed with 4% PFA for 20 min and permeabilized with 0.1% Triton X-100 for 20 min. Prior to antibody incubation, cells were blocked with 5% BSA for 1 h. Cells were then incubated with Alexa Fluor 594-conjugated CD43 antibody in 5% BSA at 4 °C overnight. After washes with PBS for 3 times, the nuclei were stained with DAPI for 10 min before observation. The stained cells were assessed with a fluorescence microscope (Nikon).

### Quantitative real-time PCR

RNA was extracted with Trizol according to the manufacturer’s instructions. RNA was transcribed into cDNA using random primers. Real-time PCR was performed on a Q6 Real-Time PCR cycler. Relative quantification of transcript levels was calculated using CT values normalized to ACTIN. Sequences for various primer pairs are shown in Supplementary Table S3.

### CHIP-qPCR

1 × 10^7^ CD31^+^ cells derived from H1 hESCs were collected for CHIP-qPCR assay. ChIP assay was conducted using the Magna ChIP™ A/G kit (Millipore) according to the manufacturer’s instructions. SMAD2/3 antibody was bought from CELL SIGNALING TECHNOLOGY (#3102S), and MSX2 antibody was purchased from SANTA CRUZ (Sc-15396). And primers used in CHIP-qPCR were listed in Supplementary Table S4.

### RNA-SEQ and bioinformatics analysis

RNA-SEQ analysis was performed by BGI Company (BGI, Shenzhen, China) as previously described [31]. Heatmap was generated using R language based on the value of log_10_ FPKM. Gene set enrichment analyses (GSEA) were performed using the GSEA software. The potential upstream TF prediction was performed using the online tool Enrichr (http://amp.pharm.mssm.edu/Enrichr/). The data are available at Gene Expression Omnibus (GEO) (Accession number: GSE134908 and GSE135171).

### Statistical analysis

Three independent experiments were performed for each analysis. All graphs depict mean ± SD. Statistical analysis was performed using Student’s *t* test to compare the difference between the two groups. The results were considered statistically significant when *P* value was less than 0.05. The graphs and statistical analyses were performed with the GraphPad Software.

## Results

### MSX2 is suppressed upon inhibition of TGFβ signaling

To explore the molecular mechanism by which TGFβ signaling regulates EHT during hematopoietic differentiation of hESCs, we added SB431542, a well-established TGFβ inhibitor, at the stage of EHT in a chemically defined hematopoietic differentiation system previously established by us [31], and subsequently performed RNA-seq by collecting the CD31^+^ cells at day 8 of differentiation (Fig. 1a). Gene set enrichment analysis (GSEA) of the data showed a marked downregulation of TGFβ signaling-associated genes in the SB431542-treated group (Fig. 1b), thus validating that inhibition of TGFβ signaling was effective. Interestingly, we found that TGFβ inhibition led to significantly elevated expression of a number of hematopoiesis-associated genes, such as GATA1, MPL, and MYB (Fig. 1c). In accordance, GSEA also demonstrated enrichment of hematopoiesis-related gene sets in the SB431542-treated cells when compared with DMSO-treated cells (Fig. 1d). These results are consistent with earlier findings that TGFβ inhibition elevates the generation of HPCs [16–18], therefore validating the reliability of our screening strategy.

To discover crucial transcription factors mediating the function of TGFβ signaling in hematopoiesis, we selected the transcription factors significantly downregulated in SB-treated cells, leading us to discover 27 genes (Fig. 1e, Fig.S1A). To narrow down the candidates, we next analyzed the transcription factors potentially acting upstream of SB431542-repressed genes during the stage of EHT using the online tool Enrichr (Fig. 1e, Fig. S1B). By combining these two strategies, we identified 4 final candidates that may mediate the function of TGFβ signaling during hematopoiesis (Fig. 1e).

Among the four candidate genes, MSX2 drew our attention due to its fundamental role in hESC mesoderm induction—the initial stage of hematopoietic differentiation [33]. In line with these observations, the mRNA expression level of *MSX2* after SB treatment was reduced by over 50%, as revealed by real-time RT-PCR analysis, while activation of TGFβ signaling by TGFβ1 treatment increased the expression of *MSX2* (Fig. 1f–g). Furthermore, CHIP-qPCR analysis revealed that there was a strong binding of SMAD2/3 to the promoter of MSX2 (Fig. 1h), suggesting that TGFb signaling directly targets MSX2 via SMAD2/3. In addition, we enriched the population of hESCs, APLNR^+^ mesoderm cells, CD31^+^CD34^+^ HEPs, and CD43^+^ HPCs, respectively, as confirmed by expression of characteristic genes for each population (Fig.S1C), and subsequently analyzed the dynamics of *MSX2* expression during hematopoietic differentiation. As previously reported [33], *MSX2* exhibited a marked upregulation during mesoderm induction from hESCs. In contrast, *MSX2* expression was rapidly reduced from HEPs to HPCs (Fig. 1i). These data suggest that MSX2 may serve as a key regulatory molecule and a potential target of the TGFβ signaling pathway during human hematopoietic differentiation.

### MSX2 deletion enhances HPC generation from hESCs

**Fig. 2:**
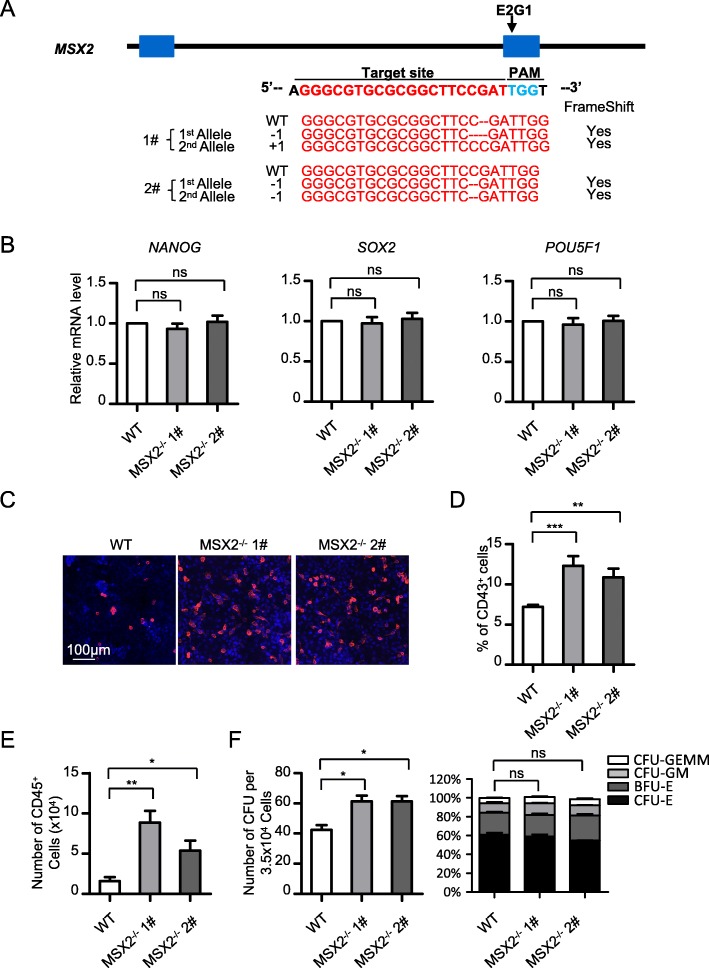
MSX2 deletion enhances the generation of HPCs from hESCs. **a** Scheme of sgRNA design and the sequences targeting exon2 of *MSX2* mediated by CRISPR/Cas9. The lower panel shows DNA sequencing results of the targeted exon of MSX2 in H1 MSX2^−/−^ 1# and 2# cells. Numbers indicate the change of nucleotides. **b** The real-time PCR analysis of *NANOG*, *SOX2*, and *OCT4* expression in undifferentiated H1 WT, H1 MSX2^−/−^ 1# and 2# cells. Expression is normalized to the level (= 1) of mRNA in WT H1 cells. **c** Representative immunofluorescence images of H1 WT and H1 MSX2^−/−^ cells showing the generation of CD43^+^ HPCs (red) at day 8 of hematopoietic differentiation. Nuclei were stained with DAPI (blue). **d** Flow cytometry analysis of the percentage of CD43^+^ HPCs from H1 WT and H1 MSX2^−/−^ cells at day 8 of hematopoietic differentiation. **e** Flow cytometry analysis showing the number of CD45^+^ hematopoietic cells from H1 WT and H1 MSX2^−/−^ cells at days 8 + 6 of hematopoietic differentiation. **f** Left panel: Total colony number generated from WT or MSX2^−/−^ H1-derived cells from chemical defined hematopoietic differentiation. Right panel: The distribution of different colony types generated from WT or MSX2^−/−^ H1-derived cells from chemical defined hematopoietic differentiation. CFU-GEMM (colony-forming unit-granulocyte/erythroid/macrophage/monocyte), CFU-GM (colony-forming unit-granulocyte/macrophage), BFU-E (burst-forming unit-erythroid), and CFU-E (colony-forming unit-erythrocyte) were documented and calculated. Results are shown as means ± SD (*n* = 3). NS, not significant; *P < 0.05, **P < 0.01, and ***P < 0.001

To study the function of MSX2 during hematopoietic development, we established MSX2-deleted hESCs by utilizing the CRISPR-Cas9 technology. We designed sgRNAs targeting the second exon of MSX2 and successfully generated two cell clones with MSX2 homozygous deletion. Frameshift and deletion of the MSX2 gene was verified by DNA sequencing (Fig. 2a). Under pluripotency culture conditions, the expression of pluripotency markers *NANOG*, *SOX2*, and *POU5F1* exhibited little difference in H1 cells with or without MSX2 knockout (Fig. 2b), indicating minimal effects of MSX2 deletion on hESC pluripotency. Therefore, these cell lines could be used for subsequent studies of MSX2 function during hematopoietic differentiation of hESCs.

By inducing hematopoietic differentiation of wild-type (WT) and MSX2-deleted cells, we observed a drastic elevation of emergent CD43^+^ hematopoietic progenitor cells from MSX2^−/−^ hESCs, as assessed with immunofluorescence analyses (Fig. 2c). When quantified with flow cytometry, the CD43^+^ hematopoietic progenitor cells exhibited a 2-fold increase with MSX2 deletion (Fig. 2d). Consistently, the number of CD45^+^ hematopoietic cells was markedly elevated in MSX2^−/−^ cells when compared with WT cells (Fig. 2e). To further evaluate the differentiation potential of generated hematopoietic progenitors after depletion of MSX2, we then performed colony formation unit (CFU) assay. The total colony number showed a significant increase with MSX2 deletion, while no significant difference was observed with regard to the distribution of different colony types (Fig. 2f). Therefore, our results indicate that MSX2 deletion enhances the generation of HPCs from hESCs.

### MSX2 deletion augments hematopoietic differentiation of hESCs by facilitating EHT

**Fig. 3:**
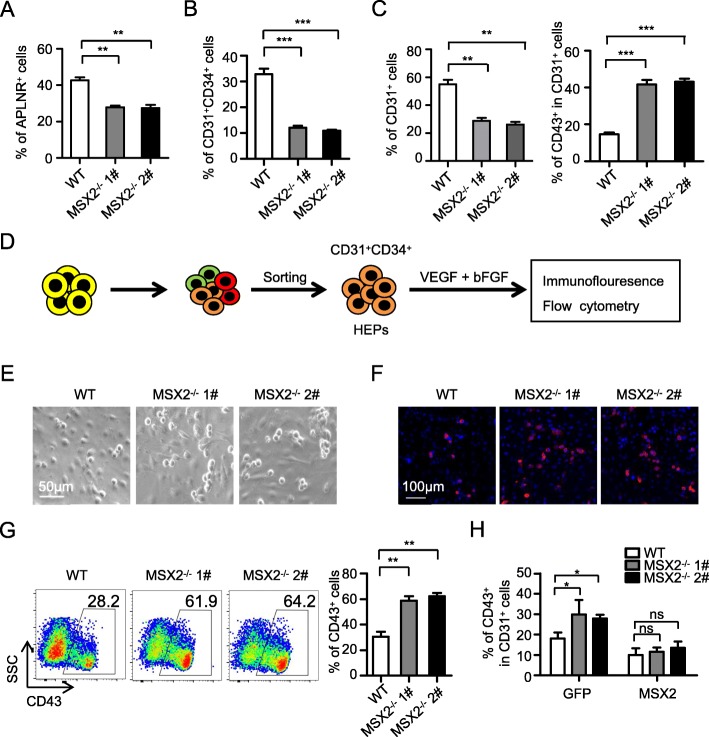
MSX2 deletion augments hematopoietic differentiation of hESCs by facilitating EHT. **a** Flow cytometry analysis of the percentage of APLNR^+^ mesoderm cells from H1 WT and H1 MSX2^−/−^ cells at day 2 of hematopoietic differentiation. **b** Flow cytometry analysis of the percentage of CD31^+^CD34^+^ HEPs from H1 WT and H1 MSX2^−/−^ cells at day 5 of hematopoietic differentiation. **c** Flow cytometry analysis showing the percentage of CD31^+^ cells (left) and CD43^+^ subpopulation gated on CD31^+^ cells (right) from H1 WT and H1 MSX2^−/−^ cells at day 8 of hematopoietic differentiation. **d** Schematic overview showing the experimental design to determine the hematopoietic potential of CD31^+^CD34^+^ HEPs. HEPs were sorted at day 5 of hematopoietic differentiation and seeded into the hematopoietic culture for 3 days before immunofluorescence and flow cytometry analysis. **e** Representative photomicrographs of cobblestone-like cells generated from H1 WT and H1 MSX2^−/−^ HEPs. **f** Representative immunofluorescence images of CD43^+^ HPCs (red) generated from H1 WT and H1 MSX2^−/−^ HEPs. Nuclei were stained with DAPI (blue). **g** Representative flow cytometry dot plots (left) and statistical analysis (right) showing the generation of CD43^+^ HPCs emerging from H1 WT and H1 MSX2^−/−^ HEPs. **h** Flow cytometry analysis showing the percentage of CD43^+^ subpopulation gated on CD31^+^ cells from H1 WT and H1 MSX2^−/−^ cells with or without MSX2 overexpression at day 8 of hematopoietic differentiation. Results are shown as means ± SD (*n* = 3). *P < 0.05, **P < 0.01, and ***P < 0.001

We showed previously that generation of HPCs from hESCs undergoes a sequential process, including mesoderm induction, HEP emergence, and HPC derivation [5]. To dissect the stage(s) at which MSX2 deletion acts to facilitate hPSC hematopoietic differentiation, we first measured the generation of early-stage cell populations, including APLNR^+^ mesoderm cells and CD31^+^CD34^+^ HEPs. We found that MSX2 deletion caused the number of APLNR^+^ cells to substantially decrease (Fig. 3a), which suggested impaired mesoderm induction and was consistent with previous findings [33]. Expectedly, the subsequent generation of HEPs was also impaired, as shown by a decrease of CD31^+^CD34^+^ cells at day 5 of differentiation (Fig. 3b). However, despite the reduction in CD31^+^ cells at day 8 of differentiation, there was still a significant elevation in the percentage of CD43^+^ subpopulation in CD31^+^ cells, indicating that MSX2 deletion could promote differentiation at the stage of EHT (Fig. 3c).

To specifically address the function of MSX2 in EHT, we enriched CD31^+^CD34^+^ HEPs from day 5 of hematopoietic differentiation and further induced the cells to undergo hematopoietic differentiation (Fig. 3d). As expected, more cobblestone-like cells were produced from cells with MSX2 deletion than those from WT cells (Fig. 3e). Consistent with the morphological changes, more CD43^+^ HPCs were generated from MSX2^−/−^ HEPs, as revealed by immunofluorescence analysis (Fig. 3f). Subsequent experiments with flow cytometry showed an over 2-fold increase in CD43^+^ HPCs derived from MSX2^−/−^ HEPs (Fig. 3g), suggesting that the MSX2^−/−^ HEPs have higher hematopoietic potential. Consistent with these observations, the hematopoietic potential of MSX2^−/−^ APLNR^+^ mesoderm cells was also enhanced (Fig. S2A-S2B).

To further confirm that the enhancement of hematopoietic differentiation was caused by the loss of MSX2, we determined whether MSX2 ectopic expression could rescue the increase of hematopoietic cells caused by MSX2 deletion. As shown in Fig. 3h, MSX2 overexpression nearly completely blocked the increase of CD43^+^ HPC generation by MSX2 depletion. Thus, although MSX2 deletion impairs mesoderm induction, it substantially promotes EHT and thereby augments the final generation of HPCs from hESCs.

### MSX2 deletion elevates EHT signature gene expression

**Fig. 4:**
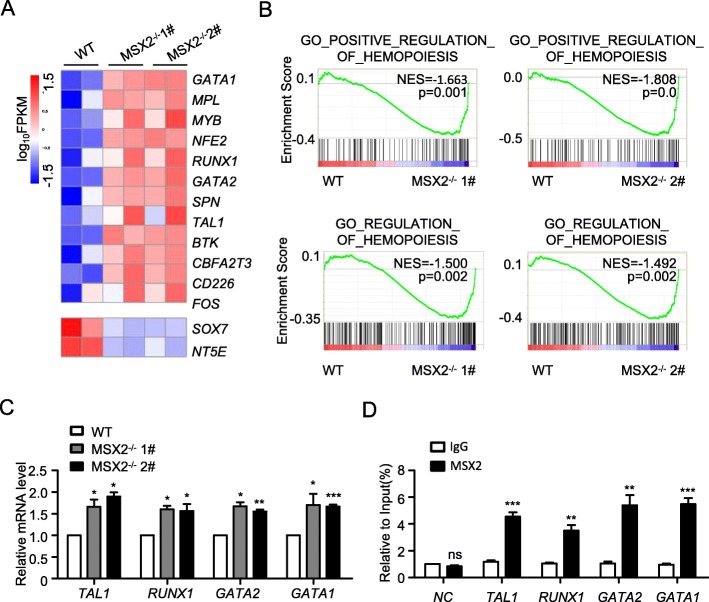
MSX2 deletion promotes upregulation of EHT signature genes. **a** Heatmap of hematopoietic signature genes in CD31^+^ cells derived from H1 WT, H1 MSX2^−/−^ 1# and 2# cells. **b** GSEA of hematopoiesis-associated gene sets in CD31^+^ cells derived from H1 WT, H1 MSX2^−/−^ 1# and 2# cells. **c** The real-time PCR analysis of *RUNX1*, *GATA2*, *TAL1*, and *GATA1* expression in CD31^+^ cells derived from H1 WT, H1 MSX2^−/−^ 1# and 2# cells at day 8 of hematopoietic differentiation. Expression is normalized to the level (= 1) of mRNA in H1 WT cells. **d** ChIP-qPCR analysis of MSX2-responsive elements on promoters of several EHT-associated transcription factors in H1-derived cells. Non-specific IgG was used as isotype control. All values are normalized to that of their corresponding input samples. Results are shown as means ± SD (*n* = 3). NS, not significant; *P < 0.05, **P < 0.01, and ***P < 0.001

To dissect the molecular mechanism underlying MSX2 regulation of EHT, we performed RNA-seq analysis using the CD31^+^ cells produced from both WT and MSX2^−/−^ hESCs to compare the gene expression profiles. In keeping with the elevated generation of HPCs with MSX2 deletion, a number of hematopoiesis-associated genes were upregulation in MSX2^−/−^ cells (Fig. 4a). Among them, a number of known regulatory genes of EHT, such as *RUNX1*, *GATA2*, and *TAL1*, were identified (Fig. 4a). Similarly, the results from GSEA also revealed the enrichment of hematopoiesis-associated gene sets in MSX2^−/−^ cells when compared with WT cells (Fig. 4b). In accordance with the bioinformatics analysis, representative genes associated with EHT, such as *RUNX1*, *GATA2*, and *TAL1*, were significantly upregulated upon MSX2 deletion, as shown by real-time RT-PCR analysis (Fig. 4c). Meanwhile, we further examined whether MSX2 were capable of binding to the promoter region of these four genes by CHIP-qPCR. Indeed, our results indicated there was a strong binding of MSX2 to the promoters of these genes (Fig. 4d). BMP, WNT, and FGF signaling play a key role in hematopoietic differentiation of hESCs [16]; we asked whether MSX2 deletion affect these pathways. However, we found that MSX2 deletion had no effect on Wnt, BMP, and FGF pathways, as assessed with GSEA (Fig.S3A-C). Thus, MSX2 deletion augments the expression of EHT signature genes, which might serve as the molecular basis for endothelial cell differentiation into hematopoietic lineages.

### MSX2 mediates the function of TGFβ signaling during EHT

What is the functional relationship between MSX2 and TGFβ signaling? We found that TGFβ inhibition results in the downregulation of MSX2 during hematopoiesis (Fig. 1f). In addition, MSX2 deletion promotes hematopoietic differentiation by facilitating EHT, mimicking the effects of inhibition of TGFβ signaling (Fig. 3d–g). These results led us to propose that MSX2 acts as a downstream mediator of TGFβ signaling.

**Fig. 5:**
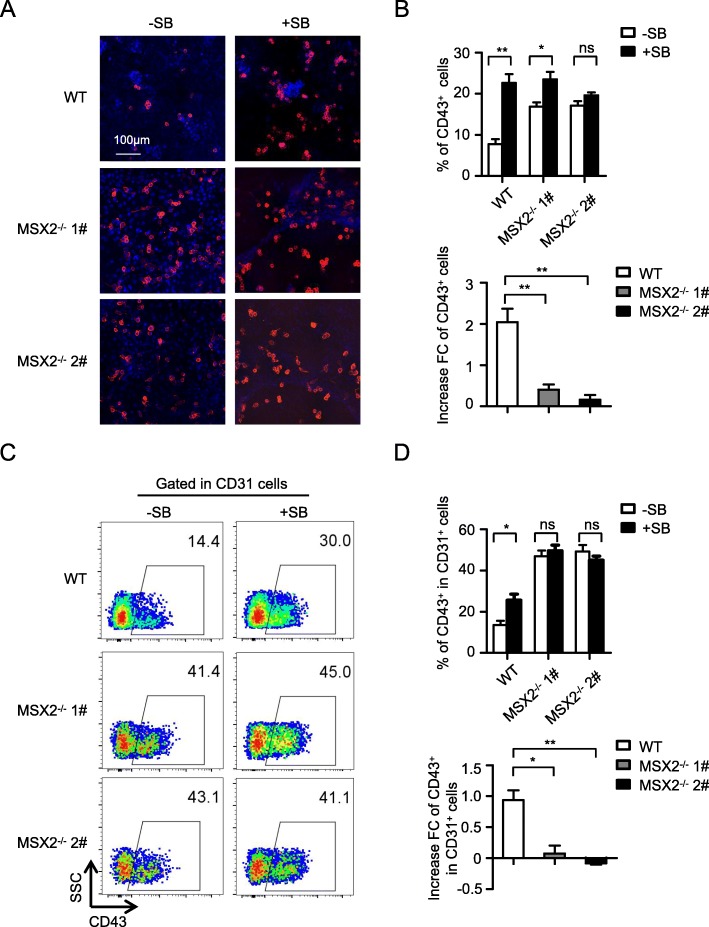
MSX2 mediates the function of TGFβ signaling during EHT. **a** Representative immunofluorescence images of CD43^+^ HPCs (red) generated from H1 WT and H1 MSX2^−/−^ cells with or without SB treatment. Nuclei were stained with DAPI (blue). **b** Upper panel: Flow cytometry analysis showing the percentage of CD43^+^ cells from H1 WT and H1 MSX2^−/−^ cells with or without SB treatment at day 8 of hematopoietic differentiation. Lower panel: The fold increase of CD43^+^ cell generation from H1 WT and H1 MSX2^−/−^ cells after SB treatment. **c** Representative flow cytometry dot plots showing the generation of CD43^+^ subpopulation gated on CD31^+^ cells from H1 WT and H1 MSX2^−/−^ cells at day 8 of hematopoietic differentiation with or without SB treatment. **d** Flow cytometry analysis showing the percentage of CD43^+^ subpopulation gated on CD31^+^ cells from H1 WT and H1 MSX2^−/−^ cells at day 8 of hematopoietic differentiation with or without SB treatment. The fold increase is also shown (lower panel). Results are shown as means ± SD (*n* = 3). NS, not significant; *P < 0.05 and **P < 0.01

To confirm this notion, we compared the effects of MSX2 deletion alone and dual treatments of MSX2 deletion and TGFβ inhibition. As depicted earlier, SB431542 treatment caused the production of CD43^+^ HPCs to elevate (Fig. 5a). Interestingly, MSX2 deletion failed to further enhance CD43^+^ HPC production in the presence of SB431542, which was further confirmed by quantitative analysis using flow cytometry (Fig. 5b, upper). Indeed, the increased fold change of CD43^+^ HPCs in MSX2^−/−^ cells with SB431542 treatment reduced from 2 (for SB431542 alone) to near 0 (Fig. 5b, lower).

We next studied the relationship between MSX2 and TGFβ signaling during EHT by determining the generation of CD43^+^ HPCs from gated CD31^+^ cells. As expected, MSX2 deletion failed to further augment the emergence of CD43^+^ HPCs from CD31^+^ cells with SB431542 treatment (Fig. 5c and Fig. 5d, upper), while the fold increase in HPC generation induced by SB431542 treatment decreased from 1 to near 0 with MSX2 deletion (Fig. 5d, lower).

To confirm these results, we utilized a previously described MSX2 inducible overexpression stable hESC lines to induce MSX2 ectopic expression at the stage of EHT [33]. Indeed, MSX2 overexpression nearly completely blocked the increase of HPC generation by SB431542 treatment (Fig.S4A). In addition, enforced expression of MSX2 robustly suppressed the increased generation of CD43^+^ HPCs from CD31^+^ cells by SB treatment (Fig.S4B). As expected, TGFβ activation by TGFβ1 treatment severely impaired the generation of CD43^+^ HPCs (Fig.S4C). However, MSX2 deletion was suffice to block the suppression of TGFβ1 on HPC generation from HEPs (Fig.S4D). To provide the direct evidence that MSX2 mediates the function of TGFβ signaling during EHT, we further established MSX2 inducible knockdown hESC lines (Fig.S4E). Flow cytometry analysis showed that MSX2 inducible knockdown during EHT process markedly attenuated the inhibitory effect of TGFβ1 on HPC emergence from HEPs by TGFβ1 (Fig.S4F and S4G). Thus, MSX2 mediates the inhibitory function of TGFβ signaling during EHT.

## Discussion

In this study, we identified MSX2 as a novel regulator of human hematopoiesis. MSX2 deletion promotes hematopoietic differentiation of hESCs by facilitating EHT. Furthermore, MSX2 acts as a downstream effector of the TGFβ signaling pathway during EHT. Therefore, our results improve the current understanding of human hematopoiesis and may therefore facilitate the development of novel strategies for efficient production of functional blood cells from hPSCs for regenerative medicine.

MSX2 participates in the development of multiple organs, such as craniofacial tissues, limb, heart, and neural crest derivates [28–30]. Nagel et al. reported that MSX2 was identified as a physiological NK-like subfamily of homeobox gene (NKL) involved in T cell differentiation via regulation of NOTCH3 signaling [34]. However, the role of MSX2 in early hematopoiesis remains to be elucidated. In this study, we showed that loss of MSX2 augments hematopoietic differentiation and therefore revealed, for the first time, the function of MSX2 in hematopoiesis. Previous studies have demonstrated that MSX2 plays important roles in mesendoderm induction [33], neural crest induction, and mesenchymal stem cell (MSC) generation from hPSCs [34]. Thus, we extend previous studies by establishing the functional role of MSX2 in hPSCs undergoing hematopoietic differentiation. Although our findings revealed that MSX2 deletion augments hematopoietic differentiation of hPSCs, no apparent defects in hematopoietic development has been observed in Msx2 knockout mice. The discrepancy between the human and the mouse studies might be because of the species difference. Similar species-specific functions of other genes have been reported previously. For example, it was reported that PAX6 is a vital determinant of neuroectoderm cell fate in hPSCs but is not required for mouse neuroectoderm specification [35]. Despite the severe hemorrhaging phenotype in Meis2-deficient mice, MEIS2-deleted hPSCs can normally differentiate into megakaryocytes and produce platelets [22].

As a vital stage of hematopoiesis, EHT is under precise and highly coordinated control of multiple transcription factors, including positive regulators (e.g., RUNX1, GATA2, and TAL1) and negative regulators (e.g., HOXA3 and SOX17) [6, 7]. In this study, we identified MSX2 as a novel negative regulator of EHT. Specifically, we detected a drastic reduction of MSX2 expression during EHT, while MSX2 deletion markedly enhances the generation of HPCs from HEPs, suggesting negative regulation of EHT by MSX2. We also showed that MSX2 deletion leads to the upregulation of several regulatory molecules, including RUNX1, GATA2, and TAL1, further confirming that MSX2 functions as a key suppressor of EHT. In keeping with our results, MSX2 has been widely regarded as a transcription repressor [36]. However, how MSX2 interacts with those EHT-regulatory factors needs to be further studied. Moreover, it was recently shown that combining one or more EHT regulators with other hematopoiesis-related genes is sufficient to directly convert somatic cells into hematopoietic cells [6, 27]. Thus, it will be of great interest to investigate whether MSX2 deletion facilitates the reprogramming processes in future studies, which may offer new strategies for the generation of functional blood cells.

Inhibition of TGFβ signaling is essential for the transition from endothelium to hematopoietic cells [16–18]. Nevertheless, how TGFβ signaling functions to block EHT has not been defined. Through genome-wide transcriptome analysis, we identified MSX2 as a transcription factor that mediates the function of TGFβ signaling during EHT. First, inhibition of TGFβ signaling leads to the downregulation of MSX2, suggesting that MSX2 acts as a downstream target of the TGFβ pathway. Furthermore, MSX2 deletion promotes hematopoietic differentiation by facilitating EHT, mimicking the effects of inhibition of TGFβ signaling Moreover, MSX2 deletion fails to further enhance the effect of TGFβ signaling inhibition on hematopoietic differentiation. Thus, our results provide new mechanistic insights into how TGFβ signaling functions to regulate EHT.

It was previously shown that TGFβ controls the development of the caudal region of the skull by targeting Msx2 [37]. Thus, this signaling axis might function in broader biological contexts. The detailed mechanisms underlying the crosstalk between TGFβ signaling and MSX2 remains to be explored. Moreover, we had shown earlier that induction of MSX2 expression by BMP signaling is essential for mesoderm production of hPSCs [33], while the current study demonstrated that MSX2 downregulation induced by inhibition of TGFβ signaling is required for EHT. Thus, temporal regulation of MSX2 by various signaling pathways during distinct stages of hematopoietic differentiation might serve as a key regulatory mechanism during the transition from hPSCs to the hematopoietic fate.

## Conclusions

In summary, we have shown that MSX2 acts as a novel regulator of human hematopoiesis and that MSX2 deletion promotes hematopoietic differentiation by facilitating EHT. Furthermore, MSX2 functions to mediate the TGFβ signaling pathway during the EHT stage. As such, our results have improved the current understanding of human hematopoiesis and may facilitate the development of novel strategies for efficient production of functional blood cells from human pluripotent stem cells (hPSCs) for regenerative medicine.

## Supplementary information

**Additional file 1 **: **Figure S1.** MSX2 is suppressed upon inhibition of TGFβ signaling during hematopoietic differentiation of hESCs. (A) Heatmap showing down-regulated TFs upon SB treatment. (B) TFs that were predicted to be upstream of the suppressed hematopoiesis-associated genes. The size and color of the bubble indicate combined score and *p*-value of the predicted term. (C) The real-time PCR analysis of the signature genes of each population in undifferentiated hESCs, MEs (APLNR+), HEPs (CD31 + CD34+) and HPCs (CD43+) generated from hESCs after hematopoietic differentiation. Relative expression is normalized to the level (=1) of undifferentiated hESCs. Results are shown as means ±SD (*n* = 3). **Figure S2.** MSX2 deletion augments the hematopoietic differentiation of hESCs. (A) Schematic overview showing the experimental design to determine the hematopoietic potential of APLNR^+^ mesoderm cells. APLNR^+^ cells were sorted at day 2 of hematopoietic differentiation and seeded into the hematopoietic culture for 5 days before CD43 flow cytometry analysis. (B) Flow cytometry analysis showing the generation of CD43^+^ HPCs emerging from H1 WT and H1 MSX2^−/−^ APLNR^+^ cells. **Figure S3.** MSX2 deletion had no effect on BMP, WNT and FGF signaling. (A) GSEA of WNT signaling between WT and MSX2^−/−^ cells. (B) GSEA of BMP signaling between WT and MSX2^−/−^ cells. (C) GSEA of FGF signaling between WT and MSX2^−/−^ cells. **Figure S4.** MSX2 mediates the function of TGFβ signaling during EHT. (A) Left panel: Flow cytometry analysis showing the percentage of CD43^+^ cells from H1 cells with or without MSX2 overexpression in the absence or presence of SB-431542 at day 8 of hematopoietic differentiation. Right panel: The fold increase of CD43^+^ cell generation from H1 cells with or without MSX2 overexpression after SB treatment. (B) Left panel: Flow cytometry analysis showing the percentage of CD43^+^ subpopulation gated on CD31^+^ cells from H1 cells with or without MSX2 overexpression in the absence or presence of SB-431542 at day 8 of hematopoietic differentiation. Right panel: The fold increase of CD43^+^ subpopulation generation gated on CD31^+^ cells from H1 cells with or without MSX2 overexpression after SB treatment. (C) Left panel: Flow cytometry analysis showing the percentage of CD43^+^ cells from H1 WT and H1 MSX2^−/−^ cells with or without TGFβ1 treatment at day 8 of hematopoietic differentiation. Right panel: The fold increase of CD43^+^ cell generation from H1 WT and H1 MSX2^−/−^ cells after TGFβ1 treatment. (D) Left panel: Flow cytometry analysis showing the percentage of CD43^+^ subpopulation gated on CD31^+^ cells from H1 WT and H1 MSX2^−/−^ cells with or without TGFβ1 treatment at day 8 of hematopoietic differentiation. Right panel: The fold increase of CD43^+^ subpopulation generation gated on CD31^+^ cells from H1 WT and H1 MSX2^−/−^ cells after TGFβ1 treatment. (E) Real-time PCR analysis of *MSX2* in H1 hESCs expressing ishMSX2–1, ishMSX2–2 or expressing a scramble shRNA (ishScramble) after the addition of DOX (2 μg/ml) during the transition from HEP to HPCs. All values are normalized to the level (= 1) of mRNA in H1 hESCs expressing a scramble shRNA (ishScramble). (F) Left panel: Flow cytometry analysis showing the percentage of CD43^+^ cells from H1 ishScramble and H1 MSX2-knockdown cells after the addition of DOX (2 μg/ml) during the transition from HEP to HPCs with or without TGFβ1 treatment. Right panel: The fold increase of CD43^+^ cell generation from H1 ishScramble and H1 MSX2-knockdown cells after TGFβ1 treatment. (G) Left panel: Flow cytometry analysis showing the percentage of CD43^+^ subpopulation gated on CD31^+^ cells from H1 ishScramble and H1 MSX2-knockdown cells after the addition of DOX (2 μg/ml) during the transition from HEP to HPCs with or without TGFβ1 treatment. Right panel: The fold increase of CD43^+^ subpopulation generation gated on CD31^+^ cells H1 ishScramble and H1 MSX2-knockdown cells after TGFβ1 treatment.

**Additional file 2 **: **Supplementary Table S1- S5**. **Supplementary Table S1**: The sequences for CRISPR sgRNAs and genotyping primers. **Supplementary Table S2**: The source of fluorochrome-conjugated antibodies used in flow cytometry. **Supplementary Table S3**: The primers used for real-time PCR. **Supplementary Table S4**: The primers used for CHIP-qPCR. **Supplementary Table S5**: RNA-seq of CD31^+^ endothelial cells derived from WT and MSX2^−/−^ hESCs.

## Data Availability

All data generated or analyzed during this study are included in this published article and its supplementary information files. Meanwhile, the datasets used and analyzed during the current study are also available from the corresponding author on reasonable request.

## Abbreviations

EHT: Endothelial-to-hematopoietic transition
GSEA: Gene set enrichment analyses
hESC: Human embryonic stem cell
HEPs: Hemogenic endothelium progenitors
HPCs: Hematopoietic progenitor cells
HSCs: Hematopoietic stem cells
hPSCs: Human pluripotent stem cells
MSC: Mesenchymal stem cell
MSX2: Muscle segment homeobox2
RA: Retinoic acid
WT: Wild-type
