# Correction to: MSX2 suppression through inhibition of TGFβ signaling enhances hematopoietic differentiation of human embryonic stem cells

**DOI:** 10.1186/s13287-020-01760-1

**Published:** 2020-06-17

**Authors:** Hongtao Wang, Mengge Wang, Yu Wang, Yuqi Wen, Xiaoyuan Chen, Dan Wu, Pei Su, Wen Zhou, Lihong Shi, Jiaxi Zhou

**Affiliations:** 1 State Key Laboratory of Experimental Hematology, National Clinical Research Center for Blood Diseases, Institute of Hematology & Blood Diseases Hospital, Chinese Academy of Medical Sciences & Peking Union Medical College, Tianjin, 300020 China; 2 Center for Stem Cell Medicine, Chinese Academy of Medical Sciences & Department of Stem Cells and Regenerative Medicine, Peking Union Medical College, Tianjin, 300020 China; 3 Department of Hematology, Xiangya Hospital, Central South University, Changsha, Hunan China; 4 Key Laboratory of Carcinogenesis and Cancer Invasion, Ministry of Education; Key Laboratory of Carcinogenesis, National Health and Family Planning Commission, Cancer Research Institute, School of Basic Medical Science, Central South University, Changsha, Hunan China; 5 State Key Laboratory of Experimental Hematology, National Clinical Research Center for Blood Diseases, Institute of Hematology & Blood Diseases Hospital, Chinese Academy of Medical Sciences & Peking Union Medical College, Tianjin, 300020 China; 6 Center for Stem Cell Medicine, Chinese Academy of Medical Sciences & Department of Stem Cells and Regenerative Medicine, Peking Union Medical College, Tianjin, 300020 China

**Correction to: Stem Cell Res Ther (2020) 11:147**

**https://doi.org/10.1186/s13287-020-01653-3**

The original article [1] contains errors in Figs. 4 & 5; Fig. 4A is misaligned and the top-left panel of Fig. 5A mistakenly duplicates the left panel of Fig. 2C in the original manuscript.

The corrected version of both Figs. 4A and 5A can be viewed ahead in this correction article.

**Fig. 4:**
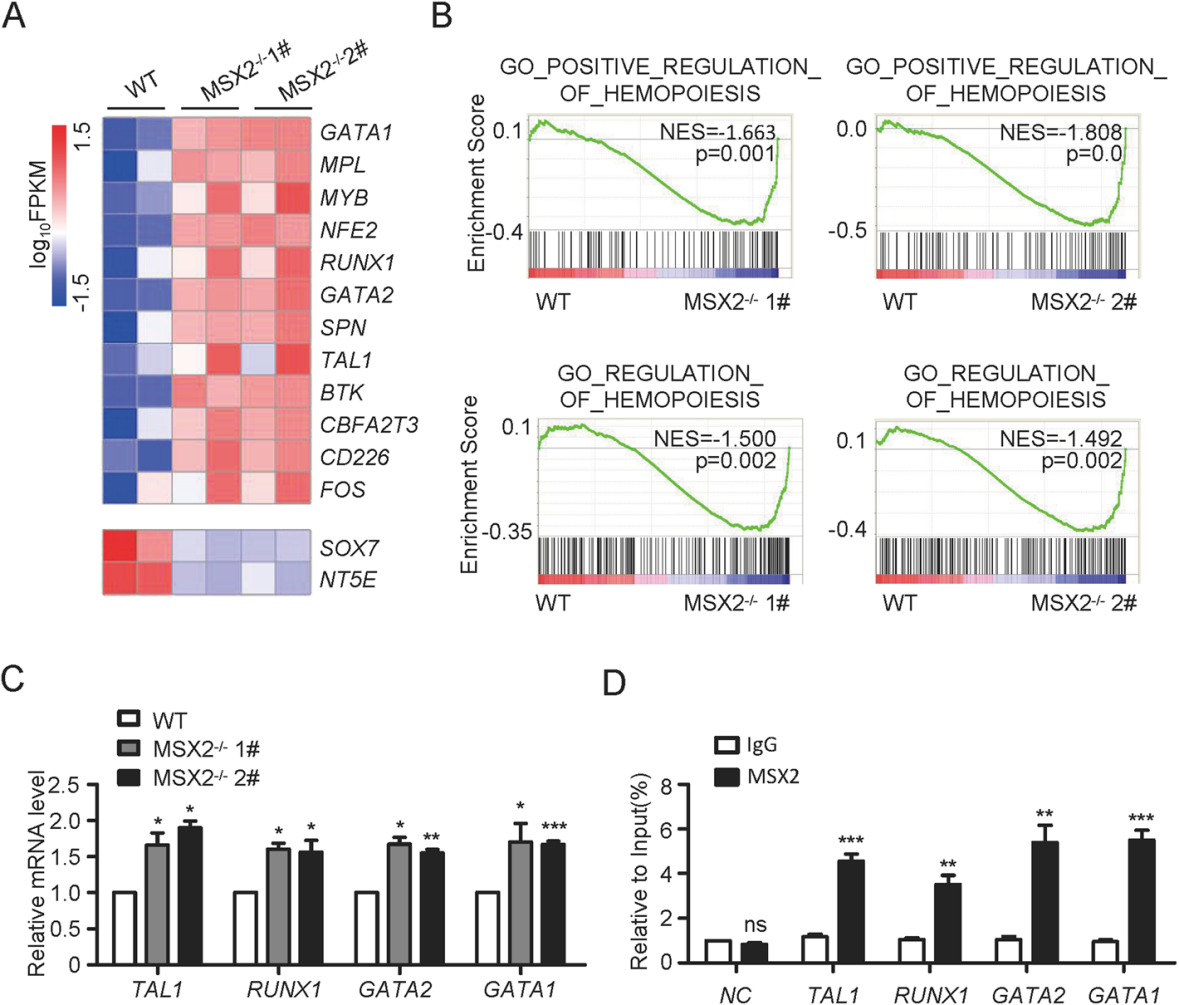
MSX2 deletion promotes upregulation of EHT signature genes. **a** Heatmap of hematopoietic signature genes in CD31^+^ cells derived from H1 WT, H1 MSX2^−/−^ 1# and 2# cells. **b** GSEA of hematopoiesis-associated gene sets in CD31^+^ cells derived from H1 WT, H1 MSX2^−/−^ 1# and 2# cells. **c** The real-time PCR analysis of *RUNX1*, *GATA2*, *TAL1*, and *GATA1* expression in CD31^+^ cells derived from H1 WT, H1 MSX2^−/−^ 1# and 2# cells at day 8 of hematopoietic differentiation. Expression is normalized to the level (= 1) of mRNA in H1 WT cells. **d** ChIP-qPCR analysis of MSX2-responsive elements on promoters of several EHT-associated transcription factors in H1-derived cells. Non-specific IgG was used as isotype control. All values are normalized to that of their corresponding input samples. Results are shown as means ± SD (*n* = 3). NS, not significant; *P < 0.05, **P < 0.01, and ***P < 0.001

**Fig. 5:**
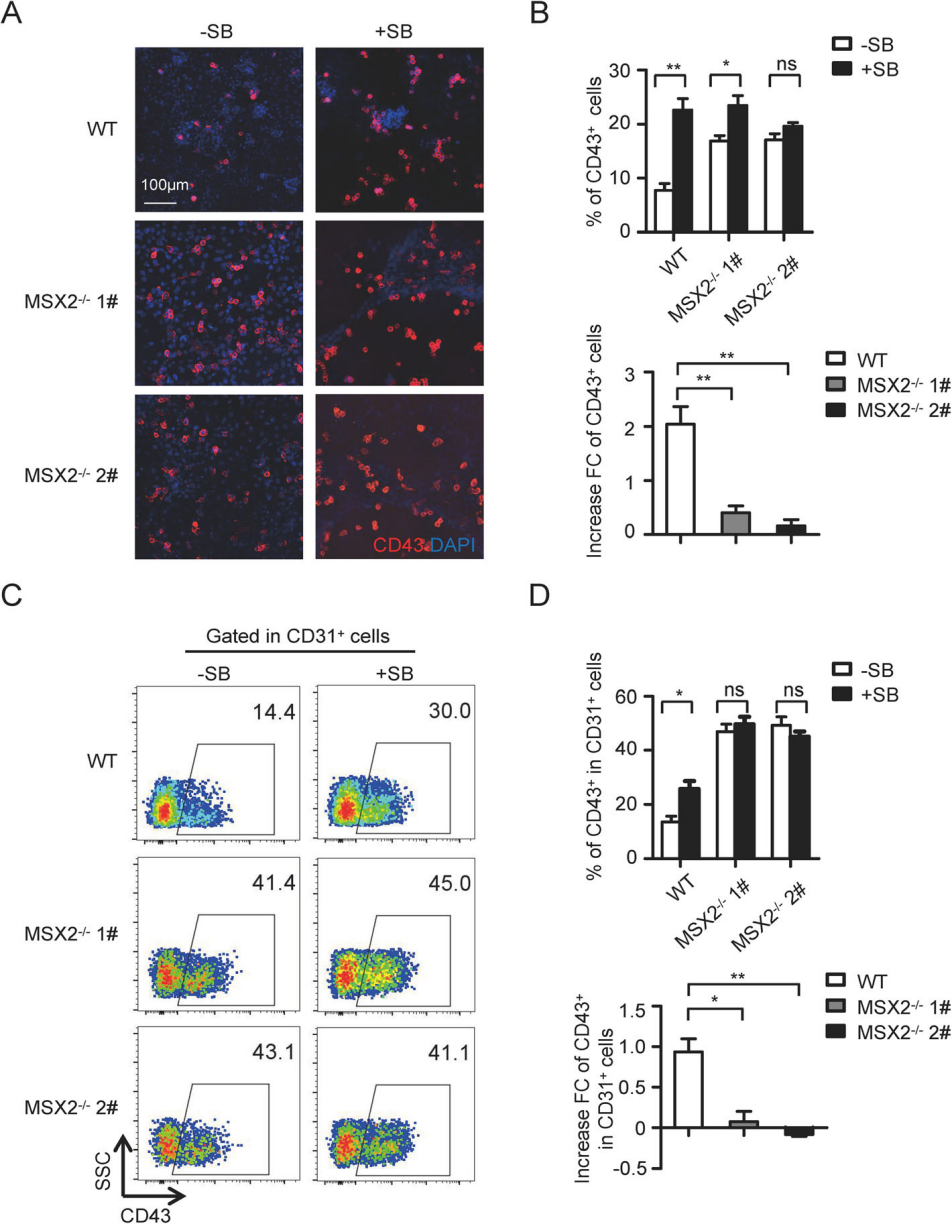
MSX2 mediates the function of TGFβ signaling during EHT. **a** Representative immunofluorescence images of CD43^+^ HPCs (red) generated from H1 WT and H1 MSX2^−/−^ cells with or without SB treatment. Nuclei were stained with DAPI (blue). **b** Upper panel: Flow cytometry analysis showing the percentage of CD43^+^ cells from H1 WT and H1 MSX2^−/−^ cells with or without SB treatment at day 8 of hematopoietic differentiation. Lower panel: The fold increase of CD43^+^ cell generation from H1 WT and H1 MSX2^−/−^ cells after SB treatment. **c** Representative flow cytometry dot plots showing the generation of CD43^+^ subpopulation gated on CD31^+^ cells from H1 WT and H1 MSX2^−/−^ cells at day 8 of hematopoietic differentiation with or without SB treatment. **d** Flow cytometry analysis showing the percentage of CD43^+^ subpopulation gated on CD31^+^ cells from H1 WT and H1 MSX2^−/−^ cells at day 8 of hematopoietic differentiation with or without SB treatment. The fold increase is also shown (lower panel). Results are shown as means ± SD (*n* = 3). NS, not significant; *P < 0.05 and **P < 0.01
