# Supplementary figures and images for: MSX2 suppression through inhibition of TGFβ signaling enhances hematopoietic differentiation of human embryonic stem cells
Source: Stem Cell Res Ther. 2020 Apr 5;11:147. doi: 10.1186/s13287-020-01653-3 (PMC7132876; doi:10.1186/s13287-020-01653-3)

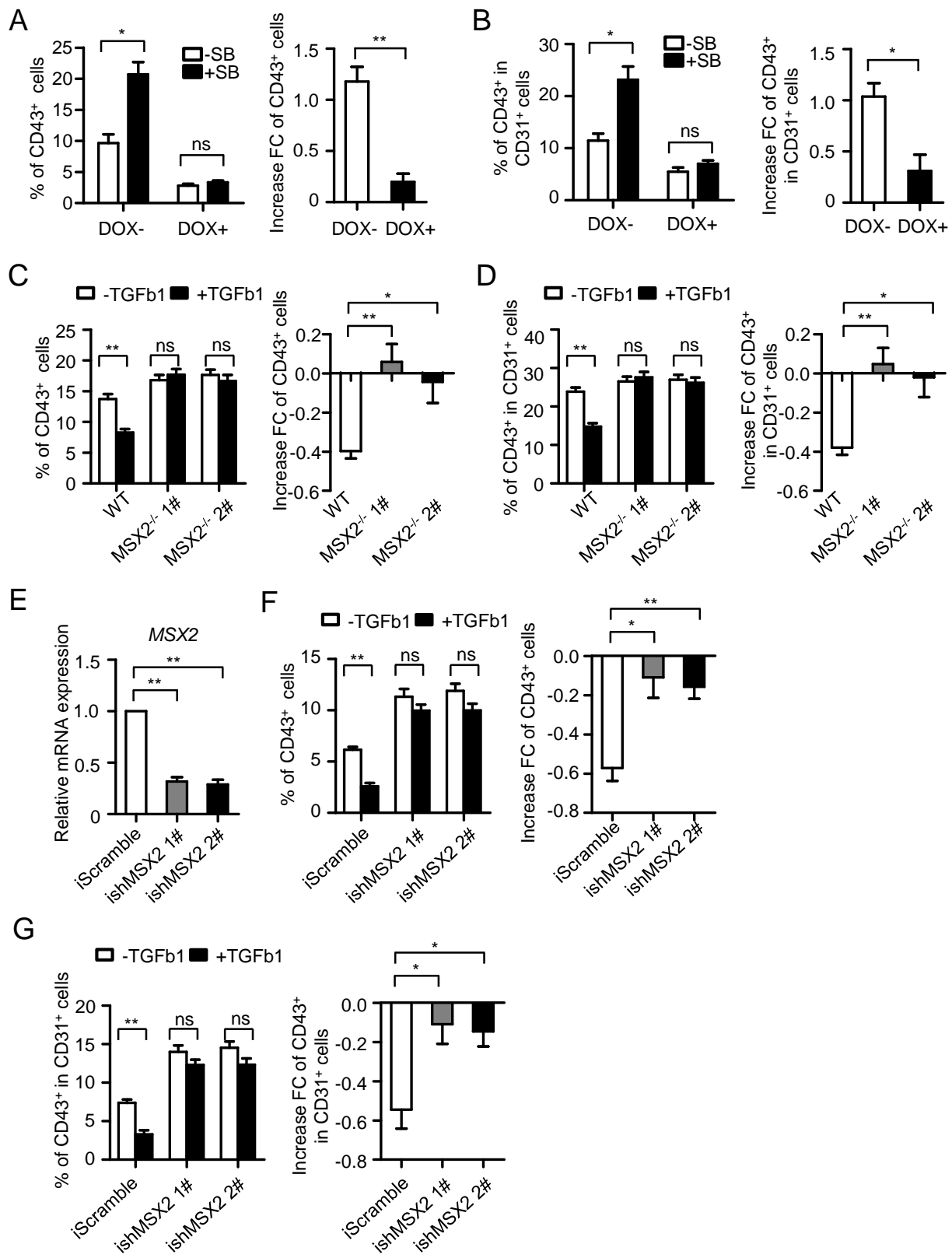

Fig.S4

Supplement: Supplementary file 1 — Additional file 1 : Figure S1. MSX2 is suppressed upon inhibition of TGFβ signaling during hematopoietic differentiation of hESCs. (A) Heatmap showing down-regulated TFs upon SB treatment. (B) TFs that were predicted to be upstream of the suppressed hematopoiesis-associated genes. The size and color of the bubble indicate combined score and p-value of the predicted term. (C) The real-time PCR analysis of the signature genes of each population in undifferentiated hESCs, MEs (APLNR+), HEPs (CD31 + CD34+) and HPCs (CD43+) generated from hESCs after hematopoietic differentiation. Relative expression is normalized to the level (=1) of undifferentiated hESCs. Results are shown as means ±SD (n = 3). Figure S2. MSX2 deletion augments the hematopoietic differentiation of hESCs. (A) Schematic overview showing the experimental design to determine the hematopoietic potential of APLNR+ mesoderm cells. APLNR+ cells were sorted at day 2 of hematopoietic differentiation and seeded into the hematopoietic culture for 5 days before CD43 flow cytometry analysis. (B) Flow cytometry analysis showing the generation of CD43+ HPCs emerging from H1 WT and H1 MSX2−/− APLNR+ cells. Figure S3. MSX2 deletion had no effect on BMP, WNT and FGF signaling. (A) GSEA of WNT signaling between WT and MSX2−/− cells. (B) GSEA of BMP signaling between WT and MSX2−/− cells. (C) GSEA of FGF signaling between WT and MSX2−/− cells. Figure S4. MSX2 mediates the function of TGFβ signaling during EHT. (A) Left panel: Flow cytometry analysis showing the percentage of CD43+ cells from H1 cells with or without MSX2 overexpression in the absence or presence of SB-431542 at day 8 of hematopoietic differentiation. Right panel: The fold increase of CD43+ cell generation from H1 cells with or without MSX2 overexpression after SB treatment. (B) Left panel: Flow cytometry analysis showing the percentage of CD43+ subpopulation gated on CD31+ cells from H1 cells with or without MSX2 overexpression in the absence or presence [file 13287_2020_1653_MOESM1_ESM.zip › Figure S4.pdf]

A

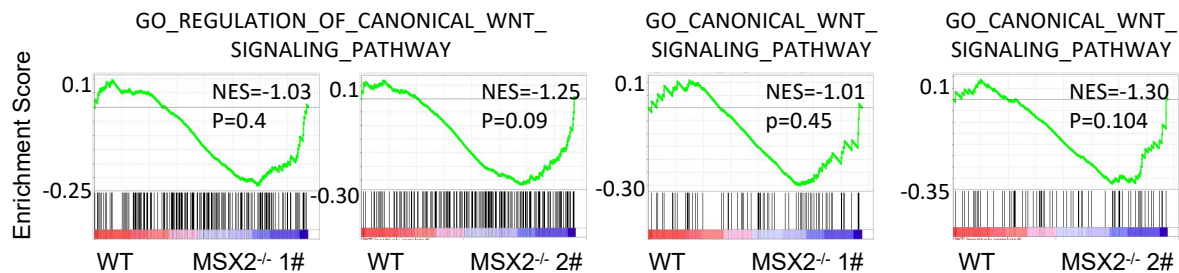

B

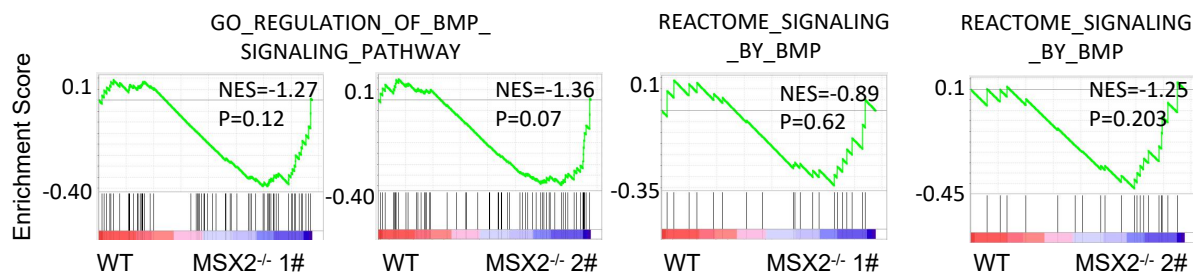

C

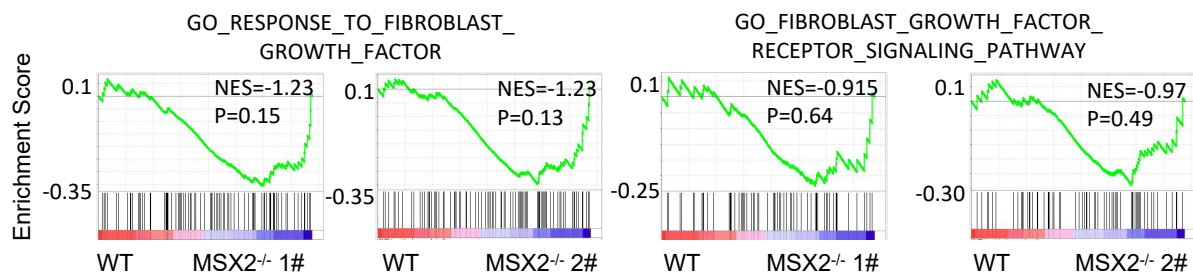

Supplement: Supplementary file 1 — Additional file 1 : Figure S1. MSX2 is suppressed upon inhibition of TGFβ signaling during hematopoietic differentiation of hESCs. (A) Heatmap showing down-regulated TFs upon SB treatment. (B) TFs that were predicted to be upstream of the suppressed hematopoiesis-associated genes. The size and color of the bubble indicate combined score and p-value of the predicted term. (C) The real-time PCR analysis of the signature genes of each population in undifferentiated hESCs, MEs (APLNR+), HEPs (CD31 + CD34+) and HPCs (CD43+) generated from hESCs after hematopoietic differentiation. Relative expression is normalized to the level (=1) of undifferentiated hESCs. Results are shown as means ±SD (n = 3). Figure S2. MSX2 deletion augments the hematopoietic differentiation of hESCs. (A) Schematic overview showing the experimental design to determine the hematopoietic potential of APLNR+ mesoderm cells. APLNR+ cells were sorted at day 2 of hematopoietic differentiation and seeded into the hematopoietic culture for 5 days before CD43 flow cytometry analysis. (B) Flow cytometry analysis showing the generation of CD43+ HPCs emerging from H1 WT and H1 MSX2−/− APLNR+ cells. Figure S3. MSX2 deletion had no effect on BMP, WNT and FGF signaling. (A) GSEA of WNT signaling between WT and MSX2−/− cells. (B) GSEA of BMP signaling between WT and MSX2−/− cells. (C) GSEA of FGF signaling between WT and MSX2−/− cells. Figure S4. MSX2 mediates the function of TGFβ signaling during EHT. (A) Left panel: Flow cytometry analysis showing the percentage of CD43+ cells from H1 cells with or without MSX2 overexpression in the absence or presence of SB-431542 at day 8 of hematopoietic differentiation. Right panel: The fold increase of CD43+ cell generation from H1 cells with or without MSX2 overexpression after SB treatment. (B) Left panel: Flow cytometry analysis showing the percentage of CD43+ subpopulation gated on CD31+ cells from H1 cells with or without MSX2 overexpression in the absence or presence [file 13287_2020_1653_MOESM1_ESM.zip › Figure S3.pdf]

A

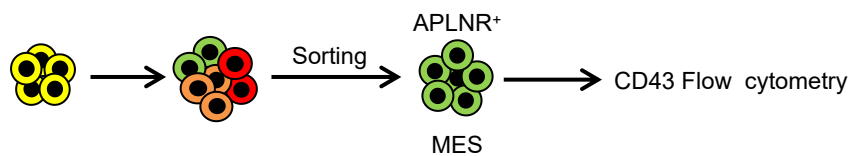

B

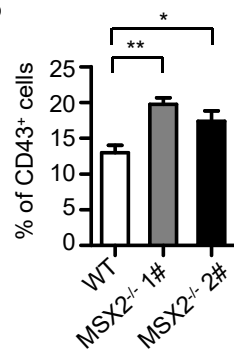

Supplement: Supplementary file 1 — Additional file 1 : Figure S1. MSX2 is suppressed upon inhibition of TGFβ signaling during hematopoietic differentiation of hESCs. (A) Heatmap showing down-regulated TFs upon SB treatment. (B) TFs that were predicted to be upstream of the suppressed hematopoiesis-associated genes. The size and color of the bubble indicate combined score and p-value of the predicted term. (C) The real-time PCR analysis of the signature genes of each population in undifferentiated hESCs, MEs (APLNR+), HEPs (CD31 + CD34+) and HPCs (CD43+) generated from hESCs after hematopoietic differentiation. Relative expression is normalized to the level (=1) of undifferentiated hESCs. Results are shown as means ±SD (n = 3). Figure S2. MSX2 deletion augments the hematopoietic differentiation of hESCs. (A) Schematic overview showing the experimental design to determine the hematopoietic potential of APLNR+ mesoderm cells. APLNR+ cells were sorted at day 2 of hematopoietic differentiation and seeded into the hematopoietic culture for 5 days before CD43 flow cytometry analysis. (B) Flow cytometry analysis showing the generation of CD43+ HPCs emerging from H1 WT and H1 MSX2−/− APLNR+ cells. Figure S3. MSX2 deletion had no effect on BMP, WNT and FGF signaling. (A) GSEA of WNT signaling between WT and MSX2−/− cells. (B) GSEA of BMP signaling between WT and MSX2−/− cells. (C) GSEA of FGF signaling between WT and MSX2−/− cells. Figure S4. MSX2 mediates the function of TGFβ signaling during EHT. (A) Left panel: Flow cytometry analysis showing the percentage of CD43+ cells from H1 cells with or without MSX2 overexpression in the absence or presence of SB-431542 at day 8 of hematopoietic differentiation. Right panel: The fold increase of CD43+ cell generation from H1 cells with or without MSX2 overexpression after SB treatment. (B) Left panel: Flow cytometry analysis showing the percentage of CD43+ subpopulation gated on CD31+ cells from H1 cells with or without MSX2 overexpression in the absence or presence [file 13287_2020_1653_MOESM1_ESM.zip › Figure S2.pdf]

A

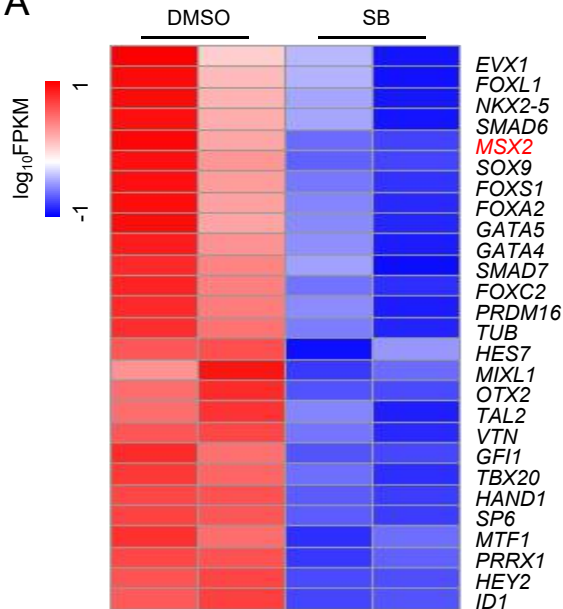

B

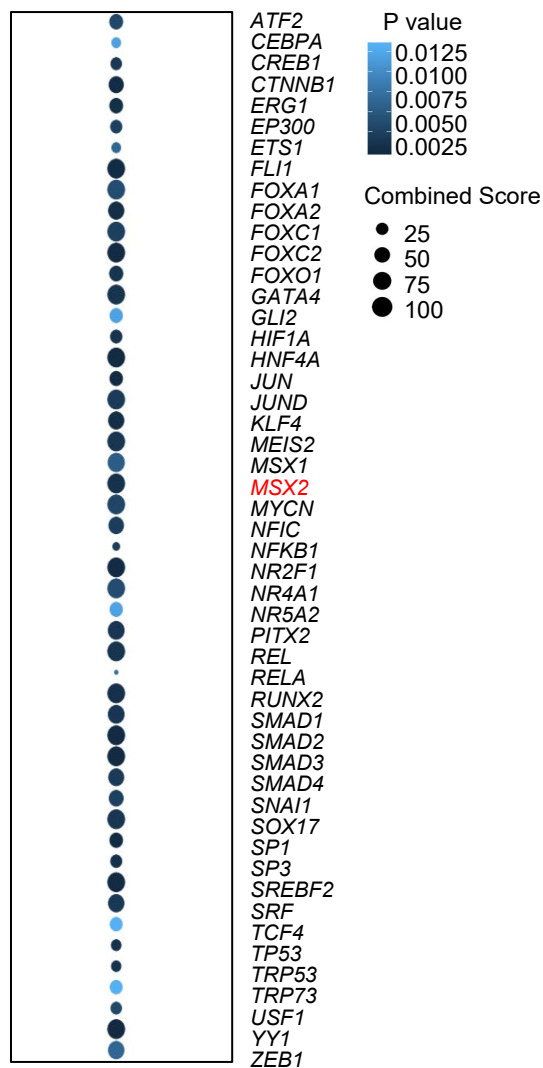

C

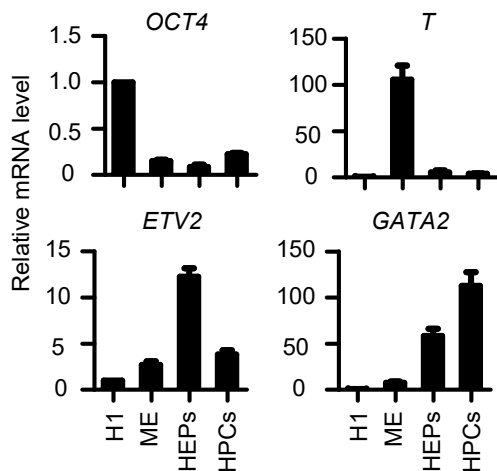

Fig. S1

Supplement: Supplementary file 1 — Additional file 1 : Figure S1. MSX2 is suppressed upon inhibition of TGFβ signaling during hematopoietic differentiation of hESCs. (A) Heatmap showing down-regulated TFs upon SB treatment. (B) TFs that were predicted to be upstream of the suppressed hematopoiesis-associated genes. The size and color of the bubble indicate combined score and p-value of the predicted term. (C) The real-time PCR analysis of the signature genes of each population in undifferentiated hESCs, MEs (APLNR+), HEPs (CD31 + CD34+) and HPCs (CD43+) generated from hESCs after hematopoietic differentiation. Relative expression is normalized to the level (=1) of undifferentiated hESCs. Results are shown as means ±SD (n = 3). Figure S2. MSX2 deletion augments the hematopoietic differentiation of hESCs. (A) Schematic overview showing the experimental design to determine the hematopoietic potential of APLNR+ mesoderm cells. APLNR+ cells were sorted at day 2 of hematopoietic differentiation and seeded into the hematopoietic culture for 5 days before CD43 flow cytometry analysis. (B) Flow cytometry analysis showing the generation of CD43+ HPCs emerging from H1 WT and H1 MSX2−/− APLNR+ cells. Figure S3. MSX2 deletion had no effect on BMP, WNT and FGF signaling. (A) GSEA of WNT signaling between WT and MSX2−/− cells. (B) GSEA of BMP signaling between WT and MSX2−/− cells. (C) GSEA of FGF signaling between WT and MSX2−/− cells. Figure S4. MSX2 mediates the function of TGFβ signaling during EHT. (A) Left panel: Flow cytometry analysis showing the percentage of CD43+ cells from H1 cells with or without MSX2 overexpression in the absence or presence of SB-431542 at day 8 of hematopoietic differentiation. Right panel: The fold increase of CD43+ cell generation from H1 cells with or without MSX2 overexpression after SB treatment. (B) Left panel: Flow cytometry analysis showing the percentage of CD43+ subpopulation gated on CD31+ cells from H1 cells with or without MSX2 overexpression in the absence or presence [file 13287_2020_1653_MOESM1_ESM.zip › Figure S1.pdf]
